# Supplementary material for: Breast Pain in a Lactating Person: An Objective Structured Clinical Examination for Clerkship Students
Source: MedEdPORTAL. 2025 Aug 22;21:11543. doi: 10.15766/mep_2374-8265.11543 (PMC12371021; doi:10.15766/mep_2374-8265.11543)
Supplement: Supplementary file 1 — SP Case.docxSP Encounter Orientation for Students.docxDoor Card.docxPostencounter Note Answer Key.docxSP Student Scoring Rubric.docxPostencounter Note Scoring Criteria.docx [file mep_2374-8265.11543-s001.zip › A. SP Case.docx]

Appendix A: *MedEdPORTAL* Standardized Patient Case

Date: 12/29/2024

Primary Case Author: Nicola Young, IBCLC

Secondary Case Author: Sarah Dotters-Katz, MD, MMHPE

Standardized Patient Educator(s): Nicola Young, IBCLC; Kelly Branford, BA

Name of Case: Breast Pain in a Lactating Person: An Observed Structured Clinical Examination for Clerkship Students

Name of Educational and/or Assessment Activity: Breastfeeding & Lactation Medicine OSCE

Patient Name: Maria Jones

Chief Complaint: “My boobs are swollen and hurt a lot.”

Most Likely Diagnosis and Differential with Rationale from History and/or Physical Exam:

1. Postpartum breast engorgement (most likely diagnosis)
   1. History: 3 days postpartum in breastfeeding female, symptoms triggered by extended period without milk removal, diffuse bilateral breast pain, difficulty latching due to swollen nipples
   2. Physical Exam: bilateral engorged breasts tender to palpation, no erythema or areas of fluctuance, afebrile, normocardic
2. Lactational mastitis
   1. History: diagnosis less likely because breast pain is not unilateral and there is no subjective fever or malaise
   2. Physical Exam: diagnosis less likely because there is no focal erythema over breast tissue, patient is afebrile, and patient is not tachycardic
3. Yeast infection
   1. History: diagnosis less likely because pain not described as burning, itching, or stinging and no history of infant oral thrush or maternal vaginal yeast infection or immunocompromise
   2. Physical exam: diagnosis less likely because nipples are not shiny/red with flaking skin, and bilateral breasts are engorged
4. Plugged duct
   1. History: diagnosis less likely because the pain is bilateral and diffuse throughout breasts, rather than in one particular area of breast
   2. Physical exam: diagnosis less likely because no palpable breast lumps/nodules, and bilateral breasts are engorged
5. Inflammatory breast cancer
   1. History: diagnosis less likely because pain is bilateral, symptoms were triggered by extended period without milk removal, and no preceding breast lump
   2. Physical exam: diagnosis less likely because no peau d’orange, breast erythema, or breast lumps

Domains: Check all that apply

- Professionalism

X. Communication and Interpersonal Skills

X. Medical History

- Physical Exam
- Shared Decision-Making

X. Patient Education

X. Clinical Reasoning

X. Documentation

- Handoff
- Presentation
- Other:

Type and Level of Learner: Undergraduate clerkship-year medical student (or more advanced)

Case Objectives: Please list specific objectives for each of the domains you have checked above:

By the end of this activity, learners will be able to:

1. Obtain and document a focused breast/chest-feeding history from a lactating parent.

2. Identify normal lactation physiology in the early postpartum period.

3. Engage a breastfeeding parent in discussion of management options for breast pain.

4. Effectively communicate with families presenting to care with infant feeding concerns.

| SETTING: outpatient, in patient, ED, home, nursing home, rehab, group, etc. | Outpatient |
| --- | --- |
| PATIENT PROFILE: Information about the “patient” that helps select an SP and helps the learner get an understanding of them as a person. SP will know more information about the patient than learner will ever ask but allows SP to portray a fully developed patient personality. If none of the items below are particulars for the case, please write “all may be used.” | |
| Age range | 30-34 years old |
| Religious/spiritual background | All may be used |
| Sex (e.g., male, female, intersex, transwoman, transman) | Female |
| Sexual orientation (e.g., heterosexual, lesbian, gay, bisexual, pansexual, queer, asexual) | Heterosexual |
| Gender expression (e.g., man, woman, genderqueer) | Woman |
| Race and ethnicity | All may be used |
| Physical description (e.g., BMI, height range) | All may be used |
| Physical limitations | None |
| Patient appearance (e.g., disheveled, hospital gown, business casual, casual) | No make-up, appearance of little sleep, wearing hospital gown |
| Moulage + location (e.g., none, bruises, scars, body piercing, tattoos) | None |
| Affect (e.g., pleasant, cooperative) | Cooperative but stressed, tired, and experiencing normal postpartum emotions |
| Family group (e.g., who is family, who they live with) | Lives with husband, 8-year-old daughter, and 3-day-old son. Has been happily married to husband for 3 years. Had first child with different partner; now separated from them. Parents living in Dominican Republic, brother is on other side of the country. Talks to family frequently but does not have large support system physically close by. |
| Education | First-generation college graduate |
| Level of health literacy | Basic health literacy |
| Employment, if any - present and past, noting any current stresses | 5^th^ grade teacher for several years |
| Home/homeless - type of dwelling, number of stories, owned or rented | Owns a small one-story home with husband |
| Financial situation - any current stresses | Lower-middle class; “My husband and I are thankfully making enough to get by right now, but if he doesn’t get his next promotion, I don’t know what we are going to do about daycare once I go back to work in 6 weeks.” |
| Insurance status (e.g., un/under/insured, public/private, HMO/PPO) | Private insurance through employer |
| Habits (i.e., diet, exercise, caffeine, smoking, alcohol, drugs) | Used to go to the gym 3x/week when her husband brought her daughter to school. Prior to the arrival of her youngest child, she would always walk their dog after work. One cup of coffee per day. 2 glasses of wine/week, none since getting pregnant. No other drugs. No tobacco use, but husband does smoke cigarettes outside of home; he has been trying to quit since Maria got pregnant. Maria tries to eat heathy. Knows that her diet could be better, especially after being diagnosed with gestational diabetes (GDM). But sometimes she feels like she doesn’t have enough time to eat as well as she knows she should. She has cut out juice and soda since being diagnosed with GDM. No dietary restrictions. |
| Activities (i.e., hobbies, sports, clubs, friends) | Loves to read and hike whenever time permits |
| Typical day - what is the usual daily routine | Everything is different now that she gave birth to second child. Prior to pregnancy and delivery, three days a week she would wake up at 5:30am, go to gym, return home and prepare daughter for school, go to work 8a-3p. Pick up daughter, return home, walk dog, make dinner, prepare for school the next day, read, watch TV or call family and go to bed by 11pm. Now she is just trying to survive the transition to caring for a newborn again. |

| CASE INFORMATION | |
| --- | --- |
| Chief Concern: What the patient will say when greeted by the student. The patient’s primary reason for seeking medical care often stated in their own words. | “My boobs are swollen and hurt a lot.” |
| Additional Concerns: Other, if any, concerns the patient has today (i.e., symptoms, requests, expectations, etc.) that will become part of set agenda. | “I’m afraid I’m not making enough breast milk to feed my baby” |
| THE PATIENT’S STORY: The SP will be asked to tell their symptom story and the personal and emotion impact for each of their concerns. You will want to write this in the patient’s voice. The symptom story should be able to answer this question: “Tell me more about [chief concern/additional concern], starting at the beginning and bringing me up to now.”  The personal context should be able to answer questions concerning the broader personal/psychosocial context of symptoms, especially the patient’s beliefs/attributions.  The emotional context should be able to ask how are you doing with this, how does this make you feel, how has this affected you emotionally? IMPACT: How has this affected your life? How has this been for your family? | “Well, my son Charlie was born 3 days ago, and we just got home from the hospital yesterday afternoon. Breastfeeding was going fine as far as I could tell. Charlie seemed to be latching well in the hospital, and I think he was cluster-feeding early last night. He then slept for 4 hours, which was awesome. But I woke up this morning and out of nowhere my breasts were so swollen and painful. I’m not sure what to do about it, and I’m afraid no milk is coming out when I feed my baby, since he has been fussy at the breast and hasn’t seemed to be latching well for the last two feeding attempts. I didn’t breastfeed with my first child, so this is all new to me. This breast pain and increasing fear that I’m not producing enough milk to feed Charlie is making me consider starting to supplement with formula or completely give up breastfeeding. I don’t know what to do at this point.” |
| HISTORY OF PRESENT ILLNESS: Although some of the HPI will be given in the patient’s symptom story, the learners will expand the story during the direct question section. Below, describe the detailed history, usually about the chief concern, which the student must develop in order to make a useful assessment of the problem: | |
| Onset (when; gradual or sudden) | Sudden onset this morning upon waking. Was comfortable when went to bed last night. |
| Setting (what was going on or where was patient when symptoms first noticed?) | Patient had finally slept for 4 hours straight since delivering her baby 3 days ago when pain started |
| Duration (how long) | Patient is seeing you 4 hours after pain onset |
| Time relationships (frequency, constant or intermittent) | Constant pain |
| Location | Both breasts, diffuse soreness throughout breasts |
| Radiation | No radiation |
| Quality | Breasts simultaneously “feel like rocks and like they are going to explode” |
| Amount | 7/10 |
| Aggravated by what | Aggravated by walking or moving around |
| Relieved by what | Temporarily relieved by a shower. Has not tried other pain relief measures outside of the scheduled acetaminophen she has been taking since delivery. Afraid that other medications might interfere with breastfeeding. |
| Associated with what | No fevers, chills, night sweats, or other associated symptoms. Patient is very tired and overwhelmed, experiencing feelings of self-doubt. |
| Attitude (what does the patient think is the problem, and how do they feel about it) | She fears she is experiencing mastitis. The whole situation is causing her a lot of self-doubt, making her feel like a failure of a parent and like her body is not sufficient to feed her child. |
| Overall course | Approximately 4 hours of constant bilateral breast pain and swelling in a lactating parent 3 days postpartum. Breast pain and swelling is complicating effective infant feeding and significantly increasing patient’s anxiety. |
| REVIEW OF SYSTEMS: Significant positives and negatives | |
| No chills (-) | **Bilateral breast pain (+)** |
| No fevers (-) | No breast lumps (-) |
| No GI, Neuro, Skin, Cardiac, or Resp symptoms (-) | No bloody nipple discharge (-) |
| Period-like vaginal bleeding (-) | **Depressed feelings (+)** |
| No dysuria, polyuria, or hematuria (-) | **Anxious feelings (+)** |
| Past medical history |  |
| Medication allergies (name and reaction) | None |
| Environmental allergies (name and reaction) | None |
| Illnesses | Major Depressive Disorder   - Well-controlled on sertraline - History of postpartum depression after birth of first child 8 years ago - No suicidal or homicidal ideation   Mild asthma   - Well-control with albuterol inhaler as needed   Diet-controlled gestational diabetes   - Diagnosed at 28 weeks |
| Vaccinations | Up to date |
| Surgeries | Cesarean section 8 years ago |
| Accidents/injuries/trauma | Broken arm after bike accident at age 10 |
| Hospitalization | Standard hospitalization after delivery of each child |
|  | |
| Inclusive sexual and reproductive history | |
| Sexual practices  Sexual partners  Protection: Use of safer sex practices  Use of birth control if appropriate  Risk of intimate partner violence | Previously sexually active with male husband, regularly engaging in vaginal and oral sex. Not currently engaging in any forms of sexual practice due to recent delivery, healing perineum, and stress/exhaustion of caring for newborn. Current form of birth control is abstinence. Low risk of intimate partner violence – patient feels safe at home. |
| OB/GYN history | Age of onset of menses – 12 years old.  Age of menopause – currently premenopausal.  Number of pregnancies – 2  Number of live births - 2  Number of miscarriages - 0  Number of abortions - 0 |
| Medications | Prescription/dose/reason   - Sertraline 25 mg daily for depression - Albuterol inhaler as needed for mild asthma   Over the counter/dose/reason   - Acetaminophen 650 mg every 6 hours as needed for mild to moderate discomfort in postpartum period   Herbs/supplements/dose/reason   - Sunflower lecithin 20g daily to try to boost milk production |
| Immunizations | X Tetanus  X. Flu  X. Hepatitis  X. Pneumovax  X HPV  X. Other: COVID-19 |
| Tobacco products:   - Cigarettes - Cigar - Pipe - Chew - E-cigarettes | X Never   - Past - year started/year quit - Current   - Quantity   - # of years |
| Alcohol   - Beer   X. Wine   - Liquor - Other | - Never - Past - year started/year quit   X. Current   - - Quantity – 2 standard drinks/week   - # of years – approximately 10 years   - None since found out she was pregnant |
| Drugs   - Weed - Cocaine - Heroin - Meth - IV - Inhalants - Other | X Never   - Past - year started/year quit - Current   - Quantity   - # of years |
| Diet (describe) | Tries to eat heathy. Knows that her diet could be better, especially after being diagnosed with gestational diabetes. But sometimes she feels like she doesn’t have enough time to eat as well as she knows she should. She has cut out juice and soda since being diagnosed. |
| Exercise (describe) | Used to go to the gym 3 times per week when her husband brought her daughter to school. Prior to the arrival of her youngest child, she would always walk their dog after work. She has not been able to do much more than a daily walk since she entered the third trimester of pregnancy. Currently adapting to life with newborn, and exercise is not a high priority. |
| List any other important social history or information important to this case | Lower-middle class: “My husband and I are thankfully making enough to get by right now, but if he doesn’t get his next promotion, I don’t know what we are going to do about daycare once I go back to work in 6 weeks.”  Husband currently trying to quit smoking cigarettes. He is only smoking outside of the home to decrease exposure for children. |
| Family history |  |
| Mother, father, siblings, grandparents, and other significant findings | Mother   - Age 65 - Breast cancer diagnosed at age 61   Father   - Age 63 - Diabetes, Type 2 diagnosed at age 52   Brother   - Age 42 - Hypertension diagnosed at age 40   Daughter   - Age 8 - No significant medical history |
|  |  |
| Physical Exam  *Vitals are provided to student at start of encounter via the Door Card.*  **Vitals**  Blood pressure: 116/72  Pulse: 88  Temperature: 98.3 F (36.8 C)  Respiration: 16  *Cardiovascular and Respiratory Exams are performed by the student during the encounter.*  **Cardiovascular Exam** – Regular rate and rhythm, no murmurs/rubs/gallops, no lower extremity edema  **Respiratory Exam** – Lungs clear to auscultation bilaterally, normal work of breathing, no wheezing  *Breast Exam Card is provided to student upon request during the encounter.*  **Breast Exam Card** – Bilateral breasts firm, swollen, and tender to palpation. Veining present bilaterally. No focal erythema over breast tissue. No masses or areas of fluctuance. Bilateral nipples edematous, no cracks or signs of bleeding. Bilateral breasts leak breast milk with hand expression. | |
| PHYSICAL EXAM FINDINGS |  |
| 1. Written in layperson’s terms | Normal physical exam apart from tired appearance and tearfulness while sharing frustrations and doubts. Breast exam card describes engorgement and tenderness to touch of both breasts. Nipples/areola are swollen but do not have cracks or bleeding. Breast milk sprays from both breasts when breasts are squeezed. |
| 1. General appearance - affect, appearance, position of patient at opening (i.e., sitting, lying down, holding abdomen, etc.) | Tired-appearing, tearful. Awake, alert & oriented to person, place, time and event. Engaging in conversation appropriately. Sitting on exam table in hospital gown. |
| 1. Vital signs | **Vitals:** Temperature 98.3 degrees F, Blood pressure 116/72, Heart rate 88, Respiratory rate 16 |
| 1. Specific findings and affect | **Cardiovascular:** Regular rate and rhythm, no murmurs/rubs/gallops, no lower extremity swelling  **Pulmonary:** Clear to auscultation bilaterally, normal work of breathing, no wheezing  **Breast Exam Card** (provided to student upon request) – Bilateral breasts firm, swollen, and tender to palpation. Veining present bilaterally. No focal erythema over breast tissue. No masses or areas of fluctuance. Bilateral nipples edematous, no cracks or signs of bleeding. Bilateral breasts leak breast milk with hand expression. |
| 1. Response to certain physical movements | N/A |
|  |  |
| DIAGNOSIS AND DIFFERENTIAL |  |
| Diagnosis with support from positive and negative history and PE findings | DIAGNOSIS:  Postpartum breast engorgement   - 1. History: 3 days postpartum in breastfeeding female, symptoms triggered by extended period without milk removal, diffuse bilateral breast pain, difficulty latching due to swollen nipples   2. Physical Exam: bilateral engorged breasts tender to palpation, no erythema or areas of fluctuance, afebrile, normocardic |
| Differential with support from positive and negative history and PE findings | Differential:   1. Lactational mastitis    1. History: diagnosis less likely because breast pain is not unilateral and there is no subjective fever or malaise    2. Physical Exam: diagnosis less likely because there is no focal erythema over breast tissue, patient is afebrile, and patient is not tachycardic 2. Yeast infection    1. History: diagnosis less likely because pain not described as burning, itching, or stinging and no history of infant oral thrush or maternal vaginal yeast infection    2. Physical exam: diagnosis less likely because nipples are not shiny/red with flaking skin, and bilateral breasts engorgement is not a manifestation of yeast infection 3. Plugged duct    1. History: diagnosis less likely because the pain is bilateral and diffuse throughout breasts, rather than in one focal area of breast    2. Physical exam: diagnosis less likely because no palpable breast lump or focal area of redness, and bilateral breasts are engorged 4. Inflammatory breast cancer    1. History: diagnosis less likely because pain is bilateral, symptoms were triggered by extended period without milk removal, and no preceding breast lump    2. Physical exam: diagnosis less likely because no peau d’orange, breast erythema, or breast lumps |
|  |  |
| MANAGEMENT OR DIAGNOSTIC PLAN | Management/Counseling:   1. Reassurance    1. *Patient is experiencing normal physiologic progression of lactogenesis II. No pathology to treat at this point, just adjust expectations and manage symptoms.* 2. Referral to lactation consultant    1. *For more support with latch, hand expression, engorgement management/prevention, hand pump use, lactation and sleep, lactation and returning to work, etc.* 3. Breast engorgement symptom management, including hand expression, reverse pressure softening of areola, NSAIDs, cold compress, regular milk removal    1. *Each of those strategies can help relieve discomfort, decrease/prevent further engorgement, and help infant latch* 4. Community resources and support groups    1. *Parent expressing doubts about ability to continue breastfeeding and some misconceptions about the realities of breastfeeding. Would likely benefit from sharing the experience with other lactating parents.* 5. Anticipatory guidance/warning signs    1. *Breast engorgement that is not appropriately managed can lead to perceived low milk supply and additional lactation challenges. Patient should be provided with warning signs for mastitis (including, but not limited to, fever, breast redness, generally feeling ill, unilateral breast tenderness/warmth to the touch) and with instruction on when to call provider/return to care.* |
|  |  |
| PROFESSIONALISM ISSUES OR CHALLENGES | None |
