# Supplementary material for: Breast Pain in a Lactating Person: An Objective Structured Clinical Examination for Clerkship Students
Source: MedEdPORTAL. 2025 Aug 22;21:11543. doi: 10.15766/mep_2374-8265.11543 (PMC12371021; doi:10.15766/mep_2374-8265.11543)
Supplement: Supplementary file 1 — SP Case.docxSP Encounter Orientation for Students.docxDoor Card.docxPostencounter Note Answer Key.docxSP Student Scoring Rubric.docxPostencounter Note Scoring Criteria.docx [file mep_2374-8265.11543-s001.zip › B. SP Encounter Orientation for Students.docx]

**Orientation to the Breastfeeding & Lactation Medicine OSCE**

Welcome to the Breastfeeding & Lactation Medicine OSCE.

All you will be permitted to bring into the patient room are your stethoscope, white coat, and a pen; no cell phones, smart watches or Maxwell Guides. All instruments you might need are already in the room. Please empty your pockets and wrists and stow these away with any other personal items.

You will participate in one station that will take a total of 25 minutes. You will have 15 minutes to take a focused history, do a physical exam, and counsel the patient on the plan of care. You will have 10 minutes to write a focused SOAP note and management plan for the patient.

You will get information about your patient and your tasks for this patient in the form of a Door Card when the session formally begins.

•    Your 15 minutes WITH YOUR PATIENT will start immediately at your station.

•    You will get an announcement after 10 minutes, that you have 5 minutes left in the room. At the end of your 15 minutes, you will hear an announcement that your time has ended. When you hear this announcement, please exit the room immediately.

•     During the 10 minutes you have for your note, you will get an announcement letting you know when you have 2 minutes left.

You don’t have to use all the time with the patient, but once you exit you cannot go back in for any reason. If you exit the room early, you can use this time to work on your patient note.

In this encounter – YOU are the provider, not ‘just a student’ on the team. Thus, you should counsel the patient, tell them the plan, explain findings or diagnoses as such.

Your physical exams will need to be very focused given the total amount of time. Forbidden physical exam maneuvers include corneal or gag reflex, breast, rectal, GU, pelvic exams or anything that would require you to remove a patient’s mask.

If you think you need to perform one of these exams, verbalize to the patient what kind of exam you’d like to perform. If there are findings, you will be handed a card. Please read and document any important information and return the card to the patient immediately. If the patient does not produce a card, you should assume all findings are normal and continue with your exam.
