# Supplementary material for: Breast Pain in a Lactating Person: An Objective Structured Clinical Examination for Clerkship Students
Source: MedEdPORTAL. 2025 Aug 22;21:11543. doi: 10.15766/mep_2374-8265.11543 (PMC12371021; doi:10.15766/mep_2374-8265.11543)
Supplement: Supplementary file 1 — SP Case.docxSP Encounter Orientation for Students.docxDoor Card.docxPostencounter Note Answer Key.docxSP Student Scoring Rubric.docxPostencounter Note Scoring Criteria.docx [file mep_2374-8265.11543-s001.zip › C. Door Card.docx]

**Patient Information**

| **Patient Name:** Maria Jones  **Setting:**  Urgent care  **Patient Information:**  Ms. Jones is a 32-year-old female 3 days postpartum presenting to urgent care for breast pain.  **Vitals:**  Blood Pressure:            116/72  Pulse:                            88  Temperature:                98.3 F (36.8 C)  Respiration:                  16 |
| --- |

**Your Task**

| In the ***15 minutes*** with the patient:              Obtain problem-focused history that includes a lactation history.  Perform the indicated physical exam.              Counsel patient on possible etiologies of their pain.  Discuss a management plan.   In the remaining ***10 minutes***             Write a note that includes a lactation history, physical exam, prioritized differential, and plan for further management. |
| --- |
