# Supplementary material for: Breast Pain in a Lactating Person: An Objective Structured Clinical Examination for Clerkship Students
Source: MedEdPORTAL. 2025 Aug 22;21:11543. doi: 10.15766/mep_2374-8265.11543 (PMC12371021; doi:10.15766/mep_2374-8265.11543)
Supplement: Supplementary file 1 — SP Case.docxSP Encounter Orientation for Students.docxDoor Card.docxPostencounter Note Answer Key.docxSP Student Scoring Rubric.docxPostencounter Note Scoring Criteria.docx [file mep_2374-8265.11543-s001.zip › D. Postencounter Note Answer Key.docx]

**Post-Encounter Note Answer Key**

| HISTORY: Describe the history you just obtained from this patient. Include only information (pertinent positives and negatives) relevant to this patient's problem(s).  CC: Breast pain  HPI: Ms. Jones is a 32 year-old G2P2 female 3 days s/p VBAC with PMHx of A1GDM and MDD who presents to urgent care with bilateral breast pain in setting of breastfeeding. Ms. Jones reports that breastfeeding was going well for the first two days, but this morning she woke up after sleeping 4 hours (her longest stretch yet since delivery) with swollen, painful breasts that “feel like they are going to explode”. She states the pain has been 7/10 bilaterally, constant for the last 4 hours, diffusely sore throughout her breasts, and non-radiating. She has been taking acetaminophen since delivery but has not used other anti-inflammatories or ice to improve the pain and has noticed that latching has been harder since symptoms started. She is not using a pump or hand expression. No cracks in nipples or bloody nipple discharge. She otherwise just feels tired and overwhelmed. Denies fever/chills, nausea/vomiting, or other infectious symptoms. She is concerned that she will not be able to continue breastfeeding.  ROS: Period-like vaginal bleeding; feeling depressed and anxious; hot flashes; fatigue. Otherwise negative apart from symptoms described in HPI.  Lactation Hx: Did not breastfeed first child (8 years ago).  PMHx: Depression, Hx PPD, mild asthma, A1GDM  Meds: Sertraline 25 mg daily, Albuterol inhaler PRN, Acetaminophen 650 mg PRN  Immunizations: up to date  PSHx: Cesarean section 8 years ago  Soc Hx: 5^th^ grade teacher, some financial worry, happily married. Rest of support system lives far away. No alcohol or tobacco use, though husband smokes outside of house.  FHx: Non-contributory | |
| --- | --- |
| PHYSICAL EXAMINATION: Describe any positive and negative findings relevant to this patient's problem(s). Be careful to include only those parts of examination you performed in this encounter.  Vitals: T 98.3C, BP 116/72, HR 88, RR 16  Gen: Tired-appearing, tearful. A&Ox4, engaging in conversation appropriately.  Breast: Bilateral breasts firm, swollen, and tender to palpation. Veining present bilaterally. No focal erythema over breast tissue. No masses or areas of fluctuance. Bilateral nipples edematous, no cracks or signs of bleeding. Bilateral breasts leak breast milk with hand expression.  CV: Regular rate and rhythm, no murmurs, rubs, or gallops, no lower extremity swelling  Pulm: Clear to auscultation bilaterally, normal work of breathing, no wheezing | |
| DATA INTERPRETATION: Based on what you have learned from the history and the physical examination, list up to 3 diagnoses that might explain this patient's complaint(s). List your diagnoses from most to least likely. Then, enter the positive or negative findings from the history and the physical examination (if present) that support or refute each diagnosis. Label positive findings with (+) and negative findings with (-). Lastly, list UP TO 5 management strategies, diagnostic studies (if any), additional orders, and/or resources you would include in your plan for this patient. | |
| Diagnosis #1: Postpartum engorgement | |
| History Finding(s) | Physical Exam Finding(s) |
| (+) 3 days postpartum in breastfeeding female | (+) Bilateral engorged breasts tender to palpation |
| (+) Symptoms triggered by extended period without milk removal | (+) No erythema or areas of fluctuance |
| (+) Diffuse bilateral breast pain | (+) Afebrile |
| (+) Difficulty latching due to swollen nipples | (+) Normocardic (no tachycardia) |
| Diagnosis #2: Lactational mastitis | |
| History Finding(s) | Physical Exam Finding(s) |
| (-) Bilateral breast pain | (-) No focal erythema over breast tissue |
| (-) No subjective fever or malaise | (-) Afebrile |
| (+) Symptoms triggered by extended period without milk removal | (-) Normocardic (no tachycardia) |
| Diagnosis #3: Yeast infection | |
| History Finding(s) | Physical Exam Finding(s) |
| (-) Pain not described as burning, itching, or stinging | (-) No shiny or flaky skin of affected nipple |
| (-) No history of infant oral thrush or maternal vaginal yeast infection | (-) Bilateral breast engorgement |
| Diagnosis #4: Inflammatory breast cancer | |
| History Finding(s) | Physical Exam Finding(s) |
| (-) Bilateral breast pain | (-) No peau d’orange |
| (-) No preceding breast lump | (-) No breast erythema |
| Diagnosis #5: Plugged duct | |
| History Finding(s) | Physical Exam Finding(s) |
| (-) Bilateral breast pain | (-) No palpable breast lump/knot |
| (+) Symptoms triggered by extended period without milk removal | (-) Bilateral breast engorgement |
| Management / Work up / Counseling | Rationale |
| Reassurance | Patient is experiecning normal physiologic progression of lactogenesis II. No need to treat at this point, just adjust expectations and manage symptoms. |
| Referral to lactation consultant | For more support with latch, hand expression, engorgement management/prevention, hands pump use, lactation and sleep, lactation and returning to work, etc |
| Breast engorgement symptom management, including hand expression, reverse pressure softening, NSAIDs, cold compress, regular milk removal | Each of those strategies can help relieve discomfort, decrease/prevent further engorgement, and help infant latch |
| Community resources and support groups | Parent expressing doubts about ability to continue breastfeeding and some misconceptions about the realities of breastfeeding. Would likely benefit from sharing the experience with other lactating parents. |
| Anticipatory guidance/warning signs | Breast engorgement that is not appropriately managed can lead to perceived low milk supply and general lactation difficulties. Patient should be provided with warning signs for mastitis (including, but not limited to, fever, breast redness, generally feeling ill, unilateral breast tenderness/warmth to the touch) and with instruction on when to call provider/return to care. |
