# Supplementary material for: Breast Pain in a Lactating Person: An Objective Structured Clinical Examination for Clerkship Students
Source: MedEdPORTAL. 2025 Aug 22;21:11543. doi: 10.15766/mep_2374-8265.11543 (PMC12371021; doi:10.15766/mep_2374-8265.11543)
Supplement: Supplementary file 1 — SP Case.docxSP Encounter Orientation for Students.docxDoor Card.docxPostencounter Note Answer Key.docxSP Student Scoring Rubric.docxPostencounter Note Scoring Criteria.docx [file mep_2374-8265.11543-s001.zip › E. SP Student Scoring Rubric.docx]

# Standardized Patient Student Scoring Rubric

**Patient's Perspective**

| Please rate how strongly you agree or disagree with the following statements: | | | | | | |
| --- | --- | --- | --- | --- | --- | --- |
|  | Strongly Disagree (1) | Disagree (2) | Neither Agree nor Disagree (3) | Agree (4) | Strongly Agree (5) | |
| Student appeared comfortable during the encounter. | ( ) | ( ) | ( ) | ( ) | ( ) | |
| Student appeared prepared during the encounter. | ( ) | ( ) | ( ) | ( ) | ( ) | |
| I was comfortable asking questions. | ( ) | ( ) | ( ) | ( ) | ( ) | |
| My personal preferences were respected. | ( ) | ( ) | ( ) | ( ) | ( ) | |
| Additional Questions | | | | | | |
| Did you understand the student's explanation for why you were experiencing the symptoms you described? | | | | ( ) No | ( )  Partially | ( ) Yes |
| Did you feel heard by the student? | | | | ( ) No | ( )  Partially | ( ) Yes |
| Did you feel as though the student understood your goals? | | | | ( ) No | ( )  Partially | ( ) Yes |

**Interpersonal**

| Interpersonal skills reflect a student’s ability to provide an effective exchange of information and develop a therapeutic relationship with their patients. They are a combination of communication skills (questioning and information-sharing) and relational skills (empathy and respect). The components of interpersonal skills being evaluated in this exam are: | | | | | |
| --- | --- | --- | --- | --- | --- |
| 9. Introduction | ( ) Poor: *Does not introduce self *Does not identify you by name *Disinterested greeting | ( )  Fair | ( ) Adequate: *Introduces self *Identifies you by name  *Appropriate greeting | ( )  Very Good | ( ) Excellent: *Introduces self by name and position  *Identifies you by name  *Warm and engaging greeting |
| 10. Questioning Skills | ( ) Poor: *No use of open- ended questions *Multiple interruptions *Scattered and disjointed line of questioning | ( )  Fair | ( ) Adequate: *Some use of open-ended questions *Few interruptions *Basic flow to line of questioning | ( )  Very Good | ( ) Excellent: *Effective use of multiple open-ended questions  *Zero to minimum interruptions *Smooth transitions and natural flow |
| 11. Elicit Patient Perspective | ( ) Poor: *Uninterested in your explanatory model  *Unconcerned with how illness may affect well-being  *Resistant to incorporating your viewpoints into decision making | ( )  Fair | ( ) Adequate:  *Acknowledges your explanatory model  *Receptive to your concerns over impact of illness on well-being *Incorporates your input into decision making | ( )  Very Good | ( ) Excellent: *Proactively assesses your explanatory model *Explores your concerns over impact of illness on well-being *Proactively solicits your viewpoint in decision making |
| 12. Verbal Communication | ( ) Poor: *Uses medical jargon excessively  *Thoughts consistently disorganized *Tone of speech conveys indifference or detachment | ( )  Fair | ( ) Adequate: *Limited use of medical jargon *Most thoughts are well organized  *Comfortable tone of speech | ( )  Very Good | ( ) Excellent: *Avoids medical jargon or readily explains it  *Thoughts are consistently well organized and easy to understand *Uses warm and accepting tone of speech |
| 13. Non-verbal Communication | ( ) Poor: *Unable to make eye contact *Awkward physical distance, facial expressions, or touching  *Conveys disinterest or apathy | ( )  Fair | ( ) Adequate: *Maintains some eye contact  *Appropriate physical distance, facial expressions, or touching *Conveys interest and concern | ( )  Very Good | ( ) Excellent: *Consistent eye contact *Uses physical distance, facial expressions, or touching effectively *Conveys attentiveness and compassion |
| 14. Empathy | ( ) Poor: *Ignores or fails to detect emotional cues  *Empathetic responses/emotional support absent or forced *Dismissive of pain or anxiety | ( )  Fair | ( ) Adequate: *Responds to emotional cues *Provides empathetic responses/emotional support  *Acknowledges pain or anxiety | ( )  Very Good | ( ) Excellent: *Perceptive of emotional cues and encourages emotional expression  *Provides empathetic responses/emotional support with genuineness and sincerity  *Attentive to pain or anxiety |
| 15. Respect | ( ) Poor: *Judgmental attitude *Makes you feel inferior *Physical exam without regard to pain or modesty | ( )  Fair | ( ) Adequate: *Non- judgmental attitude *Treats you as equal *Physical exam respectful of pain and modesty | ( )  Very Good | ( ) Excellent: *Accepting attitude *Establishes partnership *Physical exam with great sensitivity to pain and modesty |
| 16. Closure | ( ) Poor: *No explanation of impression or plan *No inquiry into remaining questions *No cordial closing remarks | ( )  Fair | ( ) Adequate: *Explains impression and plan  *Inquires about remaining questions *Cordial closing remarks | ( )  Very Good | ( ) Excellent: *Thorough discussion of impression and plan *Seeks unanswered questions, verification of understanding, and comfort level *Warm and grateful closing remarks |

**Comments**

17. Student Feedback:
