# Supplementary material for: Breast Pain in a Lactating Person: An Objective Structured Clinical Examination for Clerkship Students
Source: MedEdPORTAL. 2025 Aug 22;21:11543. doi: 10.15766/mep_2374-8265.11543 (PMC12371021; doi:10.15766/mep_2374-8265.11543)
Supplement: Supplementary file 1 — SP Case.docxSP Encounter Orientation for Students.docxDoor Card.docxPostencounter Note Answer Key.docxSP Student Scoring Rubric.docxPostencounter Note Scoring Criteria.docx [file mep_2374-8265.11543-s001.zip › F. Postencounter Note Scoring Criteria.docx]

**Scoring Criteria:**

**Pertinent History**

1) Location: bilateral, diffuse soreness throughout breasts

2) Quality: swollen – breasts firm/”feel like rocks”/“going to explode”

3) How it has affected life: starting to doubt ability to breastfeed

4) Onset: 3 days postpartum after sleeping for relatively longer stretch

5) Duration: constant since waking this morning

6) Lactation history: no history of breastfeeding with prior pregnancy

7) Birth history: vaginal delivery

8) Alleviating factors: has not pumped/hand expressed or used ibuprofen/ice

9) Associated symptoms: no subjective fevers/chills/malaise

10) Medications: sertraline and tylenol

11) Soc: supportive partner, far from majority of support system (any soc hx acceptable)

12) PMHx – gestational diabetes and major depressive disorder, history of postpartum depression

**Score for inclusion of significant information: 1 point per item, max 12

For options with multiple answers, give credit if student lists at least one

**Pertinent Physical**

1) Vitals

2) Must note that breast exam was performed

**Score for inclusion of pertinent physical exam components listed above, 2 points per item, max 4

**Differential Diagnosis**

**Leading Diagnosis (4 points)**

1) Postpartum engorgement

**Alternative Diagnoses (2 points each, maximum of 4 points)**

1. Lactational mastitis
2. Yeast infection
3. Inflammatory breast cancer
4. Plugged duct
5. Hyperlactation/Oversupply
6. Trauma

**If an alternative diagnosis is listed as the leading diagnosis, award 2 points instead of 4. If the leading diagnosis (postpartum engorgement) is listed as an alternative diagnosis, 2 points can be awarded. For example, if lactational mastitis is listed as the leading diagnosis, 2 points can be awarded. If the learner lists postpartum engorgement as an alternative diagnosis, 2 points can be awarded.

**Justification Guide**

| The table below provides the relevant data from the history and physical exam that support the leading diagnoses. Students may list other diagnoses and/or opt to include additional items.  **For every diagnosis listed by student (up to 3 maximum diagnoses), they may receive 1 point for appropriate historical data and 1 point for appropriate physical exam data if any of the data listed below is included in their justification. **Maximum of 6 points.**  Diagnosis: | **Historical Data** | **Physical Exam** |
| --- | --- | --- |
| **Postpartum engorgement** | (+) 3 days postpartum in breastfeeding female  (+) Symptoms triggered by extended period without milk removal  (+) Diffuse bilateral breast pain  (+) Difficulty latching due to swollen nipples | (+)Bilateral engorged breasts tender to palpation  (+) No erythema or areas of fluctuance  (+) Afebrile  (+) Normocardic (no tachycardia) |
| **Lactational mastitis** | (-) Bilateral breast pain  (-) No subjective fever or malaise  (+) Symptoms triggered by extended period without milk removal | (-) No focal erythema over breast tissue  (-) Afebrile  (-) Normocardic (no tachycardia) |
| **Yeast infection** | (-) No history of infant oral thrush or materal vaginal yeast infection  (-) pain not exacerbated by letdown | (-) No shiny or flaky skin of affected nipple  (-) Bilateral breast engorgement |
| **Inflammatory breast cancer**  **Plugged duct** | (-) Bilateral breast pain  (-) No preceding breast lump  (-) Bilateral breast pain  (+) Symptoms triggered by extended period without milk removal | (-) No peau d’orange  (-) No breast erythema  (-) No focal breast lump/knot  (-) Bilateral breast engorgement |

**Management / Counseling**

**Appropriate (2 points each, maximum 10 points)**

1. Reassurance
2. Referral to lactation consultant
3. Breast engorgement symptom management (must list at least one – hand expression, reverse pressure softening, NSAIDs, cold compress, regular milk removal)
4. Anticipatory guidance/warning signs (must list at least one – fever, breast redness/erythema, generally feeling ill, tachycardia, unilateral breast tenderness/warmth to the touch)
5. Community resources/support groups

**Inappropriate**

1. Antibiotics
2. Milk culture
3. Labs
