# Supplementary material for: Breast Pain in a Lactating Person: An Objective Structured Clinical Examination for Clerkship Students
Source: MedEdPORTAL. 2025 Aug 22;21:11543. doi: 10.15766/mep_2374-8265.11543 (PMC12371021; doi:10.15766/mep_2374-8265.11543)
Supplement: Supplementary file 1 — SP Case.docxSP Encounter Orientation for Students.docxDoor Card.docxPostencounter Note Answer Key.docxSP Student Scoring Rubric.docxPostencounter Note Scoring Criteria.docx [file mep_2374-8265.11543-s001.zip › _Educational Summary Report_11543.pdf]

MedEdPORTAL® The AAMC Journal of Teaching and Learning Resources

Original Publication Open Access

Breast Pain in a Lactating Person: An Objective Structured Clinical Examination for Clerkship Students

Nicola Young, IBCLC*, Kelly Branford, Deborah L. Engle, EdD, MS, Sarah K. Dotters-Katz, MD, MMHPE, Andrea Dotson, MD, MSPH, IBCLC, NABBLM-C

*Corresponding author: nryoung29@gmail.com

Abstract

Introduction: Breastfeeding provides significant health benefits for families and cost-saving potential for health care systems. Physician support is crucial to lactation success. However, lactation education curricula across the US do not reliably meet published standards, and educators lack a standardized tool to assess student competency. We developed a breastfeeding-focused OSCE for UME learners that assesses students' application of lactation knowledge in clinical settings. **Methods:** This lactation OSCE for medical students includes a patient interview, postencounter note, and evaluation by a trained standardized patient (SP). OSCE performance was compared between clerkship students who participated in a pilot lactation curriculum and students who did not participate in this curriculum. Primary outcome was correct identification of primary diagnosis. Secondary outcomes included postencounter note total score, history score, management/counseling score, and SP communication score. Data were analyzed using bivariate statistics. **Results:** Twenty-eight students completed the OSCE, of whom 15 (54%) participated in the pilot lactation curriculum. Pilot students were more likely than nonpilot students to correctly identify the primary diagnosis (73% vs. 31%, $p = .02$), score higher on management/counseling (median 4.0 [IQR: 4.0, 6.0] vs. 4.0 [IQR: 2.0, 4.0], $p = .04$), and communicate better (73% vs. 63%, $p = .002$), and also nearly twice as likely to score ≥50th percentile overall (73% vs. 39%, $p = .06$) and have higher total postencounter note scores (median 22.0 [IQR: 20.0, 26.0] vs. 19.0 [IQR: 18.0, 22.0], $p = .18$). **Discussion:** This OSCE effectively assesses UME learners' ability to clinically apply lactation knowledge.

Keywords
Breastfeeding, Lactation, Clinical Skills Assessment/OSCEs, Standardized Patient

Educational Objectives

By the end of this activity, learners will be able to:

1. Obtain a focused breastfeeding history from a lactating parent.
2. Discuss management options for breast pain with a lactating parent.
3. Effectively communicate with families presenting to care with infant feeding concerns.

Introduction

Breastfeeding—referring to any form of human milk feeding, including direct breastfeeding, chest-feeding, expressed breast milk feeding, and donor milk feeding—provides a myriad of health benefits to both infants and lactating parents.[1] These benefits include, but are not limited to, protection against infections, sudden infant death syndrome, asthma, and obesity for infants and reduced risk of certain cancers and type 2 diabetes for lactating parents.[2] However, data suggest that less than 30% of infants in the US are exclusively breastfed for the duration of time recommended by the World Health Organization (WHO).[3] Protecting and supporting breastfeeding has the potential to not only promote better health for infants and parents, but also save billions of health care dollars in its prevention of acute infection and chronic disease.[4] The low rates of breastfeeding in the US are an important public health concern that requires attention from diverse health care professionals.[5] Although lactation support providers are specifically trained to provide breastfeeding assistance, there is an insufficient number of lactation professionals to care for the quantity of lactating parents in the US and around the world. Thus, parents rely on the support of physicians and nurses for lactation success.[6]

Citation:
Young N, Branford K, Engle DL, Dotters-Katz SK, Dotson A. Breast pain in a lactating person: an objective structured clinical examination for clerkship students. *MedEdPORTAL*. 2025;21:11543.
https://doi.org/10.15766/mep_2374-8265.11543

Copyright © 2025 Young et al. This is an open-access publication distributed under the terms of the Creative Commons Attribution license.

Unfortunately, many health care staff lack the knowledge, attitudes, and skills to offer effective breastfeeding support, ultimately providing unhelpful or conflicting guidance and undermining the practice of breastfeeding.[6-8] Accordingly, there exists a fundamental need to improve competency in breastfeeding skills among health care professionals.[5] Curricula have been developed to address this knowledge gap, and those published curricula range from lectures and online modules to role plays and standardized patient (SP) workshops.[9] Most of the curricular interventions have targeted GME learners, specifically family medicine, pediatric, and OB/GYN residents, and their implementation has led to improvements in lactation care.[7] Yet, since breastfeeding skills are an important part of basic preventative health care and lactating patients may interface with many different medical specialists, all clinicians and their patients could benefit from a baseline understanding of breastfeeding.[10] In fact, the WHO, United Nations Children's Emergency Fund, US Breastfeeding Committee, Academy of Breastfeeding Medicine, and Associations of Professors of Gynecology and Obstetrics have published guidelines outlining the basic understanding of breastfeeding that clinicians from all medical specialties should possess.[10-12] Hence, UME learners, who have not yet chosen their clinical specialization, would be appropriate candidates for the introduction of lactation education.

Nevertheless, current UME students are generally not taught the recommended breastfeeding subject matter outlined by governing bodies and public health guidelines.[10] Barriers to doing so include limitations in the number of faculty knowledgeable in breastfeeding, lack of standardized breastfeeding curricula, insufficient interactions among various health care professionals, limited opportunities for practical learning, and lack of real-life experience with counseling.[13] As awareness of this curricular gap increases and programs begin to incorporate more breastfeeding education into their UME training, the lack of a consistent breastfeeding curriculum leaves room for significant variation in the training methodology. For example, some programs may incorporate breastfeeding education into their preclinical curriculum, while others may increase lactation-related clinical experiences. This variation calls for a standardized assessment tool to evaluate whether novel efforts to improve breastfeeding education are impacting students' ability to care for lactating patients.

When designing assessment tools, medical educators seek tools that are objective, comprehensive, consistent, free of bias, and designed to allow for direct observation of the examinee's skills.[14] An OSCE satisfies many of these qualities, and it can be implemented in diverse settings.[14,15] To our knowledge, an OSCE focused on assessing medical students' ability to care for lactating patients has not been published. The SP cases related to breast health that have been published are either not replicable, designed for GME learning, centered around breast cancer care, or not focused on lactation-related concerns.[16,17]

Our objective was to develop a breastfeeding-focused OSCE for UME learners. We specifically sought to evaluate whether our OSCE could effectively assess medical students' ability to clinically apply their lactation education. In designing this student assessment tool, we aimed for it to be replicable, to operate without the need for an infant or actively lactating SP, and to be applicable to the training of all UME learners.

Methods

After this project was determined to be exempt by the Duke Health Institutional Review Board (Pro00116340), a case development team, including subject matter experts from breastfeeding and lactation medicine, OB/GYN, and medical education, was assembled. We designed the SP case around a breastfeeding patient seeking care from a general practitioner in the urgent care setting, intentionally aiming for the OSCE to be relevant for all UME learners (Appendix A). To ease future implementation of the tool, we wrote the case such that it did not require an infant or an actively lactating SP. We recognize that not all lactating individuals use the term breastfeeding to describe how they feed their infants; for our purposes, we use the terms breastfeeding and lactation to refer to all forms of human milk feeding.

We developed the OSCE as a method to assess UME learners' ability to care for lactating patients. The target population for the assessment was UME students who were actively completing or had completed their clinical clerkships. Some of the students had participated in a pilot breastfeeding and lactation medicine curriculum, which included up to 12 hours of clinical lactation shadowing and access to an electronic folder with educational resources; these students are referred to as pilot students, while the remaining students, who had not participated in this pilot lactation curriculum, are referred to as nonpilot students.

We recruited participants via designated institutional email listservs for medical students who were actively participating in or had completed their clerkship year at our institution. The event was promoted as an ungraded opportunity to practice with OSCEs and support medical education research. No materials were given to learners prior to attending the session.

Copyright © 2025 Young et al. This is an open-access publication distributed under the terms of the Creative Commons Attribution license.

The primary outcome of the OSCE was documentation of the correct primary diagnosis in the postencounter note. The secondary outcomes included postencounter note total score, history score, management/counseling score, and SP communication score.

Logistics

An OB/GYN clinician-educator and a teaching assistant conducted the OSCE in a simulation center over two evenings with a total of 28 medical student participants. We instructed students to arrive at least 5 minutes before their start time. We assigned two students to each time slot, and we conducted two encounters simultaneously with two trained SPs present. Five minutes prior to each student-pair's start time, we read aloud the SP encounter orientation for students script (Appendix B). We addressed any student questions and provided them with a pen and notepad for notetaking. We then led students into the clinical skills lab, where they were assigned a room and computer station. The rooms contained an exam table, sink for handwashing, and computer for the SPs to complete the postencounter student evaluation. We gave students 15 minutes in the room with the SP, followed by 10 minutes to write a postencounter note. Bells rang to alert students at designated time points: when their time had begun; when they had 5 minutes left with the SP; when their in-person encounter time had concluded; when they had 2 minutes left to complete their note; and when the note-writing time had concluded. We utilized the clinical skills lab automated system to keep track of timing. The overall length of the encounter, including the note writing, was 25 minutes.

Students logged in to the learning space system (a secure web-based simulation center management tool [CAE Healthcare]) before entering the exam room, after the first bell rang. They were then able to see the Door Card with the instructions, which included obtaining a focused history, performing a physical exam, counseling on possible etiologies of the problem, and discussing management options (Appendix C). The Door Card also instructed students to write a postencounter note that included a lactation history, physical exam findings, prioritized differential diagnosis, and plan for further management (Appendix C). During the encounter, students received a physical exam card for any requested exam components that were forbidden. Following the encounter, they entered their postencounter note into a template within the learning space system.

After the students completed their postencounter note, we instructed them to enter a different exam room with a computer, where they filled out a brief survey that we used to collect basic background information. Following survey completion, the students departed. We then emailed students the postencounter note answer key (Appendix D).

During the implementation of the OSCE, a clinical skills lab staff member was present. They were responsible for ensuring that the learning space system was running smoothly and that the encounters remained on schedule. They were able to troubleshoot any technological challenges.

SP Training

We trained two women of reproductive age as SPs for this case. One week in advance of the training, we sent the case and scoring rubric to the SPs. We asked them to review the documents prior to the training. We held the training via video conference with the two SPs, the primary author of the case (Nicola Young), the director of the clinical skills lab, and the clinical skills lab's external client coordinator. At the start of the training, we described the purpose of the case and the motivation behind its development. We addressed the SPs' questions about the case after they reviewed it independently. We edited the case script as needed based on the SPs' feedback. During the training, each SP participated in a role play, with the primary author acting as the student conducting the encounter. Everyone who was present provided feedback and addressed any questions that came up. Each SP then practiced evaluating the student in the role play with the SP student scoring rubric (Appendix E), sharing their thought process out loud. The SPs compared their answers and discussed any ratings that differed to ensure consistency across their scoring. We reviewed any rating scale items that were unclear and corrected them immediately after the training.

Learner Assessments

Postencounter notes: As one method of student performance assessment, we scored the students' postencounter notes using the postencounter note scoring criteria (Appendix F). Two authors, who were members of the development team (Nicola Young and Sarah Dotters-Katz), scored every note and compared scores, discussing and correcting any inconsistencies.

SP student scoring rubric: After each encounter with a student, the SPs rated the student on how comfortable and confident the student appeared, how comfortable the SP felt asking questions, how well the student respected the SP's personal preferences, how well the SP understood the student's explanation of symptoms, how heard the SP felt by the student, and how well the SP felt the student understood their goals (Appendix E). The SPs also rated the students' interpersonal skills. We summed all these measures into a total SP communication score. Space was

Copyright © 2025 Young et al. This is an open-access publication distributed under the terms of the Creative Commons Attribution license.

left on the rubric for the SPs to provide additional information about students that might not have been well-reflected in the above scoring rubrics.

Statistical analysis: As data were not normally distributed, we used nonparametric bivariate statistics to analyze the data. To calculate *p* values, we utilized Kruskal-Wallis tests for continuous variables and chi-square tests for binary/categorical variables. Statistical significance was set at a *p* value of $<.05$. We analyzed the data using Stata version 18.0.

Results

Twenty-eight medical students participated in the OSCE, of whom 15 (54%) participated in the pilot lactation curriculum. The pilot students were all current clerkship-year students, while 4 (31%) of the 13 nonpilot students had completed 1 additional year of training at the time of the OSCE. The median age of participants was 26.0 years (IQR: 25.0, 27.5); age did not significantly differ between the pilot and nonpilot students ($p = .15$). Moreover, the groups did not significantly differ in personal experience with breastfeeding, confidence in supporting lactating patients prior to their clinical year of medical school, or breastfeeding education before medical school. Characteristics of all participants are provided in Table 1.

Pilot students were more likely than nonpilot students to correctly identify the primary diagnosis (73% vs. 31%, $p = .02$) and list the correct diagnosis (postpartum engorgement) in their differential (73% vs. 39%, $p = .06$; Table 2). Pilot students, compared with nonpilot students, had a higher median management/counseling score (median 4.0 [IQR: 4.0, 6.0] vs. 4.0 [IQR: 2.0, 4.0], $p = .04$) and scored higher on SP evaluation of their communication (73% vs. 63%, $p = .002$; Table 2). Furthermore, pilot students were nearly twice as likely as nonpilot students to score ≥50th percentile overall (73% vs. 39%, $p = .06$) and had higher median total postencounter note scores (median 22.0 [IQR: 20.0, 26.0] vs. 19.0 [IQR 18.0: 22.0], $p = .18$; Table 2). Median history scores were not different between pilot and nonpilot students (median 5.0 [IQR: 5.0, 7.0] vs. 6.0 [IQR: 6.0, 7.0], $p = .20$; Table 2).

Discussion

We have described the successful development of a lactation-focused OSCE for UME learners. To our knowledge, this OSCE, which assessed medical students' application of lactation knowledge in the clinical setting of a case of postpartum engorgement, is the first of its kind to be published.

We evaluated the OSCE by comparing performance between students who had participated in 12 hours of clinical lactation experience (pilot students) and students who had not received any specific lactation education in medical school (nonpilot students). Our results demonstrate that pilot students performed better on the OSCE. Specifically, pilot students were better able to identify physiologic breast engorgement in the early postpartum period, recommend appropriate management options for lactating parents experiencing breast pain, and effectively communicate with families presenting to care with infant feeding concerns. We did not observe a significant difference in history-taking abilities during the OSCE between pilot and nonpilot students. We can explain this lack of difference in part by the fact that our measure of history-taking ability relied on the documentation of the SP case history. Pilot students may have gathered a more thorough lactation history during the encounter, but this was not reflected in their postencounter note. Moreover, this OSCE was ungraded, which could have influenced student documentation detail and effort. Due to challenges with recruitment, the cohort of nonpilot students also included four students who had 1 additional year of medical school experience, while the pilot cohort contained all clerkship-year students, presenting a potential confounding factor.

Table 1. Characteristics of Student Participants in the Lactation OSCE

Characteristic	Overall (*N* = 28)	Pilot Student[a] (*n* = 15)	Nonpilot Student[b] (*n* = 13)	*p*
Demographic				
Age, *mdn* years [IQR]	26 [25.0, 27.5]	26 [25.0, 27.0]	27 [26.0, 28.0]	.15
Sex, *n* (%)				.05
Male	3 (11)	0 (0)	3 (23)	
Female	25 (89)	15 (100)	10 (77)	
Experience, *n* (%)				
Personal experience with breastfeeding	8 (29)	4 (27)	4 (31)	.81
Did not feel confident supporting breastfeeding patient prior to clerkship year	23 (82)	11 (73)	12 (92)	.19
Breastfeeding education before medical school	3 (11)	1 (7)	2 (15)	.46
Current clerkship-year student	24 (86)	15 (100)	9 (69)	.02

[a]Student who participated in pilot lactation curriculum.
[b]Student who did not participate in lactation curriculum.

Copyright © 2025 Young et al. This is an open-access publication distributed under the terms of the Creative Commons Attribution license.

Table 2. OSCE Assessment Data

Assessment Measure	Overall (N = 28)	Pilot Student[a] (n = 15)	Nonpilot Student[b] (n = 13)	p
Correct primary diagnosis, n (%)	15 (54)	11 (73)	4 (31)	.02
Correct diagnosis on differential, n (%)	16 (57)	11 (73)	5 (39)	.06
History score, *mdn* [IQR][c]	6.0 [5.0, 7.0]	5.0 [5.0, 7.0]	6.0 [6.0, 7.0]	.20
Management/counseling score, *mdn* [IQR][d]	4.0 [2.0, 5.0]	4.0 [4.0, 6.0]	4.0 [2.0, 4.0]	.04
Total postencounter note score, *mdn* [IQR][e]	21.0 [18.0, 24.5]	22.0 [20.0, 26.0]	19.0 [18.0, 22.0]	.18
≥50th percentile in total postencounter note score, n (%)	16 (57)	11 (73)	5 (39)	.06
Standardized patient communication score, *mdn* % [IQR][f]	66.5 [61.5, 75.0]	73.0 [68.0, 80.0]	63.0 [60.0, 65.0]	.002

[a]Student who participated in pilot lactation curriculum.
[b]Student who did not participate in lactation curriculum.
[c]Total possible history score = 12.
[d]Total possible management/counseling score = 10.
[e]Total possible postencounter note score = 40.
[f]Total possible standardized patient communication score = 100%.

Importantly, based on our observations, we learned that we could have enhanced the quantification of students' ability to take a history from a lactating patient by utilizing a video recording or documenting SP recall of questions asked. In comparison to a written postencounter note, these data might have allowed us to better assess students' history-taking skills. Nevertheless, we are encouraged that students with lactation education generally performed better on the OSCE, especially since the pilot curriculum was not tailored to the SP case. Though we were able to utilize the infrastructure of our institution's clinical skills lab, we believe it would be possible to conduct this OSCE in a different setting, such as a classroom, with student or faculty volunteers playing the SP role, should resources be limited.

Our OSCE, designed for UME learners, differs in the scope of its learning objectives from previously published lactation-related OSCEs that were targeted to GME learners. According to the Academy of Breastfeeding Medicine, medical students are expected to demonstrate basic skills, such as taking a breastfeeding history and recognizing common lactation issues, while residents are expected to manage complex lactation scenarios and provide comprehensive counseling.[18] Although our OSCE could also be valuable for GME learners, given their often limited prior exposure to lactation education and the significant overlap in lactation-related educational guidelines across training levels, it is designed to align with UME competencies.[19] Additionally, to our knowledge, the existing literature does not contain an OSCE focused on postpartum engorgement.

It is important to note the limitations of this lactation OSCE and its development. We implemented and evaluated it at a single medical center with a relatively small sample size that was not randomly selected, which limits generalizability. We assessed students after their participation in only one pilot curriculum; we have not yet utilized the OSCE to assess student performance following other lactation-related curricular interventions. This OSCE assesses student ability to clinically apply lactation knowledge in the setting of one SP lactation case. The participants were not all at the exact same point in their medical training; the majority were clerkship-year students, but 14% had experienced an additional year of training. The participants engaged in the OSCE anonymously, and the results had no bearing on their grades; this may have impacted the extent of student effort directed toward the postencounter note. Video footage of the encounters or SP recall of key history components would have likely enhanced evaluation of this OSCE. OSCE scores were overall low for both the pilot and nonpilot student groups, and though this is consistent with other OSCEs used at our institution, those who plan to deploy it at their institution may need to develop internal norms to align the scoring with their grading strategy.[20,21] Should this assessment tool be utilized in an educational setting in the future, we encourage incorporating time for debrief and direct student feedback on their performance.

In conclusion, this OSCE effectively assessed UME learners' ability to clinically apply lactation education. Given that the current landscape of lactation pedagogy in US UME curricula does not live up to national or international standards, this assessment tool can be a resource for evaluating educational initiatives aiming to address the gap in training.[10-12] Furthermore, this OSCE could be adapted to serve as a learning tool in addition to an assessment tool in the future.

Appendices

A. SP Case.docx

B. SP Encounter Orientation for Students.docx

C. Door Card.docx

Copyright © 2025 Young et al. This is an open-access publication distributed under the terms of the Creative Commons Attribution license.

D. Postencounter Note Answer Key.docx

E. SP Student Scoring Rubric.docx

F. Postencounter Note Scoring Criteria.docx

All appendices are peer reviewed as integral parts of the Original Publication.

Nicola Young, IBCLC: Medical Student, Duke University School of Medicine

Kelly Branford: Clinical Skills Program Director, Duke University School of Medicine

Deborah L. Engle, EdD, MS: Assistant Dean for Assessment and Evaluation, Duke University School of Medicine

Sarah K. Dotters-Katz, MD, MMHPE: Associate Professor, Department of Obstetrics and Gynecology, Duke University School of Medicine

Andrea Dotson, MD, MSPH, IBCLC, NABBLM-C: Assistant Professor, Department of Family Medicine and Community Health, Duke University School of Medicine

Disclosures
None to report.

Funding/Support
None to report.

Ethical Approval
Duke Health Institutional Review Board reviewed this project.

References

1. Victora CG, Bahl R, Barros AJ, et al; Lancet Breastfeeding Series Group. Breastfeeding in the 21st century: epidemiology, mechanisms, and lifelong effect. *Lancet*. 2016;387(10017): 475-490. https://doi.org/10.1016/S0140-6736(15)01024-7
2. Roghair R. Breastfeeding: benefits to infant and mother. *Nutrients*. 2024;16(19):3251. https://doi.org/10.3390/nu16193251
3. World Health Organization; United Nations Children's Fund (UNICEF). *Global Strategy for Infant and Young Child Feeding*. World Health Organization; 2003. Accessed June 28, 2025. https://www.who.int/publications/i/item/9241562218
4. Walters DD, Phan LTH, Mathisen R. The cost of not breastfeeding: global results from a new tool. *Health Policy Plan*. 2019;34(6): 407-417. https://doi.org/10.1093/heapol/czz050
5. Mulcahy H, Philpott LF, O'Driscoll M, Bradley R, Leahy-Warren P. Breastfeeding skills training for health care professionals: a systematic review. *Heliyon*. 2022;8(11):e11747. https://doi.org/10.1016/j.heliyon.2022.e11747
6. Rollins NC, Bhandari N, Hajeebhoy N, et al; Lancet Breastfeeding Series Group. Why invest, and what it will take to improve breastfeeding practices? *Lancet*. 2016;387(10017):491-504. https://doi.org/10.1016/S0140-6736(15)01044-2
7. Feldman-Winter L, Barone L, Milcarek B, et al. Residency curriculum improves breastfeeding care. *Pediatrics*. 2010;126(2): 289-297. https://doi.org/10.1542/peds.2009-3250
8. Gavine A, MacGillivray S, Renfrew MJ, Siebelt L, Haggi H, McFadden A. Education and training of healthcare staff in the knowledge, attitudes and skills needed to work effectively with breastfeeding women: a systematic review. *Int Breastfeed J*. 2017;12:6. https://doi.org/10.1186/s13006-016-0097-2
9. Chuisano SA, Anderson OS. Assessing application-based breastfeeding education for physicians and nurses: a scoping review. *J Hum Lact*. 2020;36(4):699-709. https://doi.org/10.1177/0890334419848414
10. Gary AJ, Birmingham EE, Jones LB. Improving breastfeeding medicine in undergraduate medical education: a student survey and extensive curriculum review with suggestions for improvement. *Educ Health (Abingdon)*. 2017;30(2):163-168. https://doi.org/10.4103/efh.EfH_180_15
11. Association of Professors of Gynecology and Obstetrics (APGO). *Medical Student Education: Guidelines for Medical Schools.* 11th ed. APGO; 2014. Accessed June 28, 2025. https://cdn.ymaws.com/resource/resmgr/apgo-11th-ed-mso-book.pdf
12. World Health Organization; United Nations Children's Fund (UNICEF). *Protecting, Promoting and Supporting Breastfeeding: The Special Role of Maternity Services. A Joint WHO/UNICEF Statement*. World Health Organization; 1989. Accessed June 28, 2025. https://iris.who.int/bitstream/handle/10665/39679/9241561300.pdf
13. Michaud-Létourneau I, Gayard M, Lauzière J, et al. Understanding the challenges related to breastfeeding education and barriers to curricular change: a systems perspective for transforming health professions education. *Can Med Educ J*. 2022;13(3):91-104. https://doi.org/10.36834/cmej.73178
14. Joshi MK, Srivastava AK, Ranjan P, et al. OSCE as a summative assessment tool for undergraduate students of surgery-our experience. *Indian J Surg*. 2017;79(6):534-538. https://doi.org/10.1007/s12262-016-1521-y
15. Patrício MF, Julião M, Fareleira F, Carneiro AV. Is the OSCE a feasible tool to assess competencies in undergraduate medical education? *Med Teach*. 2013;35(6):503-514. https://doi.org/10.3109/0142159X.2013.774330
16. Haughwout JC, Eglash AR, Plane MB, Mundt MP, Fleming MF. Improving residents' breastfeeding assessment skills: a problem-based workshop. *Fam Pract*. 2000;17(6):541-546. https://doi.org/10.1093/fampra/17.6.541
17. Karpa K, Stollar K. Rash in breast-feeding mother: standardized patient case for clerkship students. *MedEdPORTAL*. 2016;12:10453. https://doi.org/10.15766/mep_2374-8265.10453

Copyright © 2025 Young et al. This is an open-access publication distributed under the terms of the Creative Commons Attribution license.

18. Meek JY; Academy of Breastfeeding Medicine. Educational objectives and skills for the physician with respect to breastfeeding, revised 2018. *Breastfeed Med*. 2019;14(1):5-13. https://doi.org/10.1089/bfm.2018.29113.jym

19. Breastfeeding curriculum: Objective Structured Clinical Examination case study. American Academy of Pediatrics. Accessed June 28, 2025. https://www.aap.org/en/pedialink/breastfeeding-curriculum/objective-structured-clinical-examination-case-study

20. Adkins LD, Harris BS, Gesher C, et al. Understanding patient evaluation of abnormal uterine bleeding (AUB): a standardized patient case on AUB for OB/GYN clerkship students. *MedEdPORTAL*. 2022;18:11216. https://doi.org/10.15766/mep_2374-8265.11216

21. Reid HW, Branford K, Reynolds T, Baldwin M, Dotters-Katz S. It's getting hot in here: piloting a telemedicine OSCE addressing menopausal concerns for obstetrics and gynecology clerkship students. *MedEdPORTAL*. 2021;17:11146. https://doi.org/10.15766/mep_2374-8265.11146

Received: November 30, 2024
Accepted: July 8, 2025
Published: August 22, 2025

Copyright © 2025 Young et al. This is an open-access publication distributed under the terms of the Creative Commons Attribution license.
